# Adaptive Energy-Efficient Target Detection Based on Mobile Wireless Sensor Networks

**DOI:** 10.3390/s17051028

**Published:** 2017-05-04

**Authors:** Tengyue Zou, Zhenjia Li, Shuyuan Li, Shouying Lin

**Affiliations:** College of Mechanical and Electronic Engineering, Fujian Agriculture and Forestry University, Fuzhou 350002, China; chinayuyi@163.com (Z.L.); lsyfafu@163.com (S.L.); linshouying@fafu.edu.cn (S.L.)

**Keywords:** wireless sensor network, alarm, intrusive detection, energy control, data fusion

## Abstract

Target detection is a widely used application for area surveillance, elder care, and fire alarms; its purpose is to find a particular object or event in a region of interest. Usually, fixed observing stations or static sensor nodes are arranged uniformly in the field. However, each part of the field has a different probability of being intruded upon; if an object suddenly enters an area with few guardian devices, a loss of detection will occur, and the stations in the safe areas will waste their energy for a long time without any discovery. Thus, mobile wireless sensor networks may benefit from adaptation and pertinence in detection. Sensor nodes equipped with wheels are able to move towards the risk area via an adaptive learning procedure based on Bayesian networks. Furthermore, a clustering algorithm based on *k*-means++ and an energy control mechanism is used to reduce the energy consumption of nodes. The extended Kalman filter and a voting data fusion method are employed to raise the localization accuracy of the target. The simulation and experimental results indicate that this new system with adaptive energy-efficient methods is able to achieve better performance than the traditional ones.

## 1. Introduction

Wireless sensor networks (WSNs) involving dozens or even hundreds of sensor nodes powered by batteries over a large field play an increasingly important role in a wide range of detecting applications, e.g., environment monitoring, wildlife tracking, battlefield intelligence and area security [1,2,3]. With the development of automation, sensor nodes are able to work by themselves without directions from human being. Thus, they can be spread by planes or vehicles in harsh environments and report sensory data to the gateway node without men for decision making. Due to the random deployment, the locations of the nodes under the circumstances are uncertain, but the engineers usually try to make them roughly uniformly cover the area. Because the geographical condition and the position of sensoring object are specific in each environment, the locations of sensor nodes should be adjusted after initialization to acquire optimization. If there are no workers to assist in changing their positions, mobile nodes with movable equipment are good choices for these unmanned systems.

**Figure 1:**
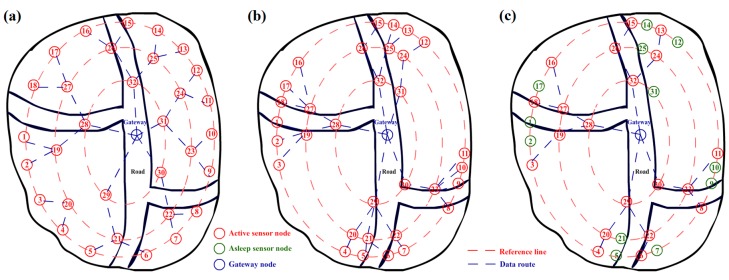
(**a**) Illustration of the initial arrangement of sensor nodes for a detection system; (**b**) illustration of the new arrangement of sensor nodes after adaptive learning and movement; and (**c**) active sensor nodes after representative nodes are selected for energy savings.

A mobile wireless sensor network (MWSN) consisting of mobile nodes is suitable to deal with the intruder detection applications in field and forest, especially under unmanned environments. The main task of these applications is to detect the presence or absence of a particular target or event in a region of interest [4]. Nodes Equipped with pyroelectric infrared (PIR) sensors are able to detect entrance of strangers or animals. The smoke and temperature sensors can be used to alarm fire in forests [5]. Detecting accuracy is an important item in this kind of applications. Because the probability of intrusion is not equally scattered among different locations in a field, the detecting sensor nodes actually do not need to cover every point of the scene equally. Thus, an adaptive deployment of a mobile wireless sensor network (MWSN) can help the system improve accuracy and reduce energy consumption [6,7]. Figure 1 shows an example of adaptive location adjusting of sensor nodes. To prevent loss of generality, scene is set in a bounded filed with a main road and two arterial roads. The roads represent the hidden paths naturally created in the area by geographical factors or animal feeding behaviors, which imply the key region needed to be focused on in the environment. Generally, most of the scenes contain this kind of regions which need more attention than other ones. Figure 1a shows the initial positions of sensor nodes in the intrusion-detecting system based on an MWSN. We assume that most of the intrusions are from those three roads in the scene. As the adaptive method designed in this work, the nodes move towards the area with high intrusive risk to build a new arrangement, as Figure 1b shows. This procedure will lead to a higher sensitivity to the target in the risk area because there will be more sensor nodes in the region. Based on the gathering, the data fusion algorithm [8,9,10] can also be further employed to increase accuracy and robustness.

Energy control is another important item in MWSN. Wireless sensor nodes are typically powered by batteries with limited capacity [11]. The lifetime of a sensor node is the time taken to discharge its battery below a level sustainable for operation [12]. Without energy harvesting equipment, energy-efficient techniques are the only way to save energy and prolong the lifetime of sensor nodes. However, the movement in MWSN will cost additional energy than stationary one. Thus, in this work, clustering and representative algorithms are introduced to reduce the number of active sensor nodes to save energy, as Figure 1c shows. Setting a learning threshold is another helpful way for reducing energy cost. After several rounds of learning and self-adjusting, the sensor nodes can be commanded to stay still and actually evolved to the stationary ones. This mechanism will help the system to keep a balance between accuracy and energy cost.

The main contributions of this work are described as follows: (1) a Bayesian network (BN) is employed to adjust the arrangement of the MWSN adaptively; (2) a data fusion method and a tracking function based on the extended Kalman filter (EKF) are introduced to improve the robustness of the system; and (3) energy-efficient methods are designed to reduce the energy consumption. The remainder of this report is organized as follows. After reviewing related studies in Section 2, an adaptive learning procedure based on a BN is presented in Section 3. Section 4 shows a data fusion method based on target tracking. Energy-efficient methods are also presented to prolong the lifetime of the system. In Section 5, simulations are performed in MATLAB to verify the effects of these algorithms. Then, experiments are conducted on real-time hardware implemented with the Texas Instruments CC2530 platform, and the results are presented. Finally, the conclusions are stated in Section 6.

## 2. Related Studies

Target detection is certainly one of the most important topics in sensor network research. In a sensor network, sensor nodes are often deployed in a region of interest to monitor the presence of a particular target or event [4]. Many technical schemes have been proposed in the literature for target detection using sensor networks [10,11,12,13,14,15]. A 2D barrier coverage method, which requires many fewer sensors than full coverage, is designed to reduce the average power consumption [10]. However, it cannot detect the targets immediately; it can sense them only before they cross the monitored field. An acoustic WSN with a specialized fusion center has been developed to detect and track multiple targets simultaneously [11]. The system is reported to have relatively low computational complexity and high overall tracking accuracy. A WSN with inexpensive sensor nodes are successfully designed in [12] for target detection, classification, and tracking. Three specific motion models are established to classify different target classes, and reliability, energy and sensoring options are also discussed in the work. However, the system has not taken the hidden regulations of geography under circumstance into consideration and the sensor nodes in it do not own the ability of movement. There is also work focusing on the implementation of a detecting and tracking system based on wireless sensor device [13]. Its detailed realization includes hardware, software, system and radio. Probabilistic methods are found to be taken for event detection or node localization. A deployment algorithm is developed using potential field to reach maximum coverage of nodes [14]. Probability distribution is also employed to navigate the sensor nodes towards the location of the alarm [15]. However, these studies have not noticed the gradually decreasing property of the alarm probability along with the spread and not taken the hidden regulations of geography into consideration. Due to the power limitation of WSNs, energy efficiency is taken into consideration. In [16], an energy-efficient distributed multisensor target detection method is proposed to save energy, and a sleep/wake-up mechanism is presented. When there are no targets in the detection area, a large number of sensors are put to sleep to reduce the power consumption. Furthermore, to save energy, a novel intelligent distributed cooperative method is designed, inspired by the regulating mechanisms of the human hormone system [17]. The algorithm enables the sensor nodes to self-organize themselves autonomously without a centralized control. Thus, the transmission load between general nodes and the specialized central node can be reduced.

A clustering approach is another way to improve the efficiency of target detection [18]. A node is elected as a clusterhead based on its highest residual energy in a zone. The clusterhead is set to be active for detection, and other nodes may remain in inactive mode to conserve energy. Some researchers consider the detection task to be an integral model involving location and communication [19]. Thus, not only the tracking algorithm but also the communication protocol is discussed. Moreover, cameras are added to the WSN to achieve wide-area video surveillance [20]. In this study, each camera node uses an adaptive Gaussian mixture model to extract moving targets and an unscented Kalman filter (KF) to track targets. In addition to a single target or multiple targets in the application environment, there are certain continuous objects such as forest fires, biochemical materials and mudflows that need to be detected. Due to their nature and characteristics of changing size and shape, they pose new challenges for the detection algorithm. A new data structure and method are proposed to solve this problem [21]. The new data structure reduces the communication cost of the overall algorithm without compromising the accuracy, and the new method achieves good performance in simulation. All the methods mentioned above are based on the fixed sensor nodes without moving equipment. Once they are deployed in the application environment according to the arrangement algorithm, they will operate until their energy is exhausted. Because the arrangement algorithm does not always obtain optimal results through early calculations, it is challenging to achieve satisfactory adaptability during target detection with fixed sensor nodes. Thus, we adopt an MWSN in this research, whose nodes can move via the motors and wheels with which they are equipped. This change improves the pertinence and adaptability of the system and is proved to be more advantageous in the experiments below.

## 3. Adaptive Learning Procedure

Because of the different geographical structures and path deployment, each place in a scene has a different chance of being intruded upon. Thus, the sensor nodes of a wireless network do not need to be uniformly arranged to cover the entire scene, and the entrances with high intrusion probability require more attention to avoid missed targets or false alarms. A BN is introduced in this work to acquire the probability of intrusion in each area, and the movement of sensor nodes is based on it. At the beginning of the system’s operation, all the sensor nodes are deployed according to an initial arrangement, and they subsequently move after each learning step to achieve optimization.

### 3.1. Initial Arrangement

**Figure 2:**
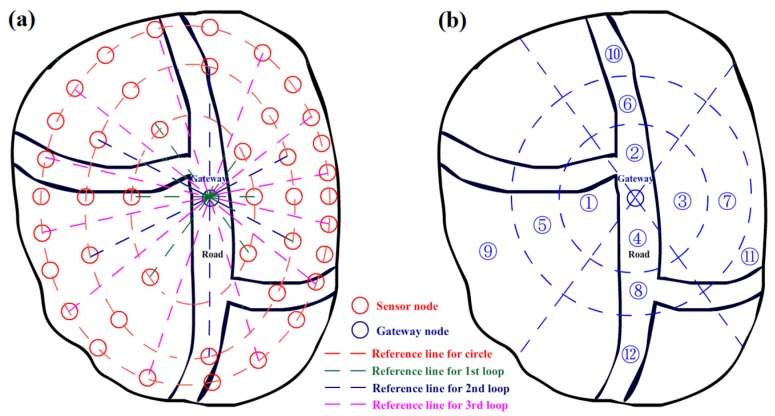
(**a**) Recommended initial arrangement of sensor nodes for a detection system; and (**b**) recommended area division method of sensor nodes for adaptive learning and movement.

There are many ways to set the initial positions for sensor nodes. If the designer does not know the key points in the area, it is suggested to place the sensor nodes uniformly over the area. In this work, we provide an advised method for addressing the problem. As shown in Figure 2a, a set of ellipses marked by a red dotted line is made as a reference line to surround the gateway node at the center. The parameter *R_int_* is chosen by the designer to indicate the number of ellipses. Then, the reference lines in the radiation direction are also made according to an equal angle value, which is decided by the sensor number parameter *D_n_*, *n =* 1, 2, 3, *…*, where *n* represents the number of loops. In the illustration of Figure 2a, *D*_1_ is set to 6, which indicates that there are six sensor nodes in the first loop; the interval angle value is 360/*D*_1_ = 60°. Thus, the reference lines for the first loop marked by the green dotted line are drawn by the angle interval of 60°. The sensor nodes of the first loop are placed at the intersection of the ellipse reference and the radiation reference of the first loop. For the second loop, *D*_2_ is set to 2 × *D*_1_, and the reference lines for the second loop marked by the blue dotted lines are added to the middle position of each interval between two neighboring reference lines of the first loop. Then, 12 sensor nodes are added to the second loop. According to the same rule, 24 sensor nodes are arranged for the third loop, as shown in Figure 2a.

After the arrangement of sensor nodes, the entire area needs to be divided into several pieces for the learning procedure. The division method can be chosen by the designer of the system. Figure 2b shows an example of the division in this work. The area is divided into twelve pieces by two concentric circles and two reference straight lines at 60° and 120°. Then, each piece of the area can be represented by a node to build a BN for the learning procedure, and an intrusion probability is assigned to each node to express its possibility of being intruded upon. Following the division, the sensor nodes in the region of each piece are set to belong to the piece and will move towards the area with a high probability of intrusion according to the learning procedure.

### 3.2. BN and Learning

Due to the uniform arrangement of sensor nodes in the initial step, each area in the field receives equal attention for intrusion. However, because of the complex environment, the intruder may avoid detection in the risk area, causing serious consequences if there are not sufficient sensor nodes. If there are only one or two sensor nodes for an important area, noise in the background may lead to them making incorrect judgments. Thus, deployment of more sensor nodes for areas of concern and fewer for peaceful areas may reduce the detection failures and improve the accuracy of the entire system. At the beginning of operation, without prior knowledge or human assistance, it is difficult for the system to discover the areas of concern with high intrusion risk. However, after the detection of intrusion, the system can focus its attention on these areas gradually by the adaptive learning procedure. A BN is a powerful tool for making probabilistic inferences on complex domains and is especially well suited for modeling this learning procedure [22,23]. A BN structure is formally composed of nodes and arcs. The nodes are used to represent random variables, and the arcs represent the influence of one node on another, which is quantified by their conditional probabilities. For example, an arc from node A to B shows the conditional dependence of B given A, which can be quantified as P(B|A). The nodes connected by an arc are called the parent node and child node. A child node may have several parent nodes, which means it is affected by several factors. In a similar manner, one parent node can have several child nodes, meaning that this factor may have an influence on several other factors. Thus, after the structure is confirmed, the BN is able to be fully described by a set of conditional probability between the nodes.

**Figure 3:**
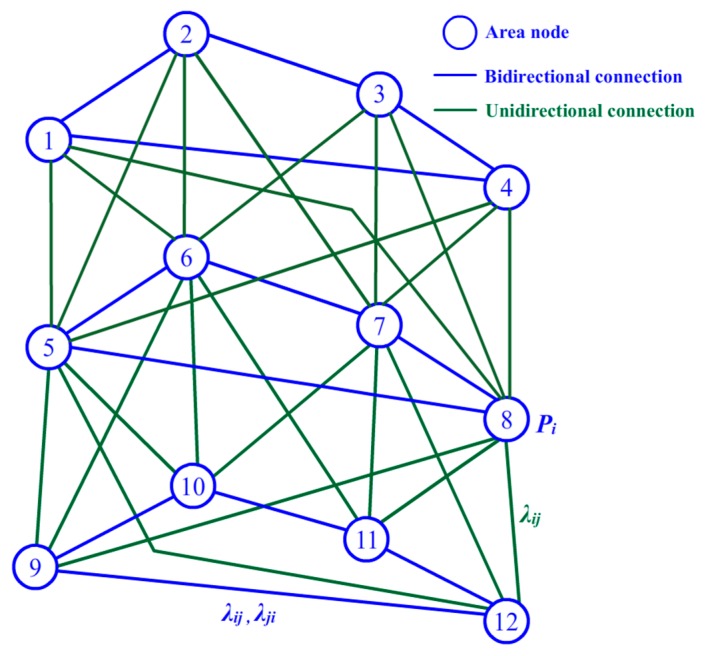
Illustration of a Bayesian network (BN) for adaptive learning.

In this work, the BN is used to model the relationship between area pieces. As shown in Figure 3, an area piece is presented by an area node according to the division in Figure 2b. Each area node is assigned a probability value noted by *P_i_* to indicate its possible degree of being intruded upon by a stranger. Because an intrusive target may enter the surrounding area in the next time interval, when a target appears in an area piece, the intrusion probability values of its node and the nodes of its neighboring area pieces should be increased together to improve the guarding capability. Thus, arcs of the BN are a suitable tool to describe these relationships. First, we can divide all the area pieces into several levels according to the concentric circles for reference. For example, as shown in Figure 2b, all the nodes can be divided into three levels from outside to inside. Nodes 9–12 are set to be the first level, and nodes 5–8 are set to be the second one. Nodes 1–4 compose the third level. Because the intrusive target approaches the center position gradually under most circumstances, we assume in our model, without loss of generality, that a target may move between two areas in the same level or from a low level to a high level from outside to inside, but cannot move from a high level to a low level. Although the system may miss some situations in which a target enters the concerning area and leaves by itself after a short period of time under this assumption, this detection failure will not be dangerous in terms of guarding of the entire area. Thus, this assumption can help us simplify the system model and conserve calculation resources. As shown in Figure 3, two types of connections are needed for our BN model: bidirectional connections marked by blue lines and unidirectional connections marked by green lines. A bidirectional connection indicates that the intrusive target is able to move from one side to the other freely, while a unidirectional connection allows movement only from low level to high. The parameters *λ_ij_* and *λ_ji_* in Figure 3 are used to indicate the probability of the corresponding movement. *λ_ij_* is assigned for the movement from node *i* to node *j*. Similarly, *λ_ji_* represents the probability from node *j* to node *i*. For example, as shown in Figure 3, node 9 has a bidirectional connection with nodes 10 and 12 on its same level, and it also has a unidirectional connection with nodes 5, 6, and 8 on a higher level. In other words, if an intrusive target already exists in the area of node 9, the system will estimate its chance of entering the surrounding areas of nodes 10 and 12 or the high level areas of nodes 5, 6, and 8.

**Figure 4:**
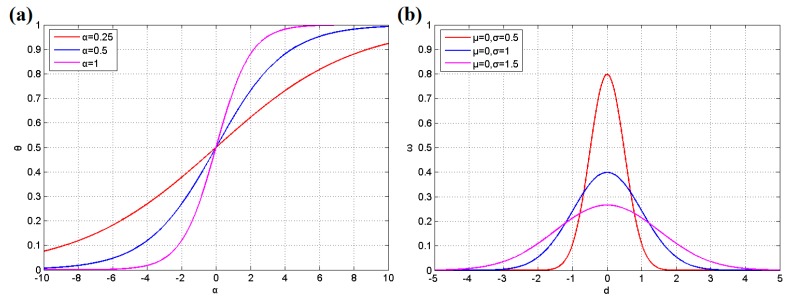
(**a**) Function graph of Equation (3); and (**b**) function graph of Equation (15).

After estimation, *P*_9_, which is the probability value of node 9, should be raised to improve the security according to Equation (1), where *P_i_* denotes the old probability value and *P_i_*’ represents the new probability value after updating; Δ*P_i_* is an incremental step parameter determined by the user that indicates the influence level of each intrusion; A larger Δ*P_i_* will increase the influence of an intrusion but reduce the chance to revise the error by subsequent learning round; *θ*(*x*) is a mapping function for adjusting the accumulative effect of the equation, which is shown in Equation (3), where *x* is the intrusion count for this area; and *η* is an adjustment coefficient for the learning speed assigned by the user. Additionally, the probability values of the areas surrounding node 9 must be increased as a precaution according to the arcs of the BN and Equation (2). Equation (2) is similar to Equation (1), and the extra parameter *λ_ij_* is used to reflect the connection between two neighboring areas. As the alarm probability decreases during diffusion, the *λ_ij_* should be set gradually lower along with the increment of distance. In practice, the value of parameter *λ_ij_* for each arc in the BN should be decided by the user following the application environment. Moreover, Figure 4a illustrates the function graph of Equation (3), and it was found that the parameter *α* influences the slope of the curve. A higher *α* value leads to sharp variation in early accumulation and the change will be slighter later. However, a low *α* value leads to relatively uniform change. It can be assigned by engineers according to the application environment.

(1) $$P_{i}^{\prime }=P_{i}+\omega \cdot (\theta (x)-\theta (x-1))\cdot \Delta P_{i}$$

(2) $$P_{j}^{\prime }=P_{j}+\omega \cdot (\theta (x)-\theta (x-1))\cdot \lambda_{ij}\cdot \Delta P_{i}$$

(3) $$\theta =\frac{1}{1+e^{-\alpha x}}$$

### 3.3. Node Movement and Target Detection

**Figure 5:**
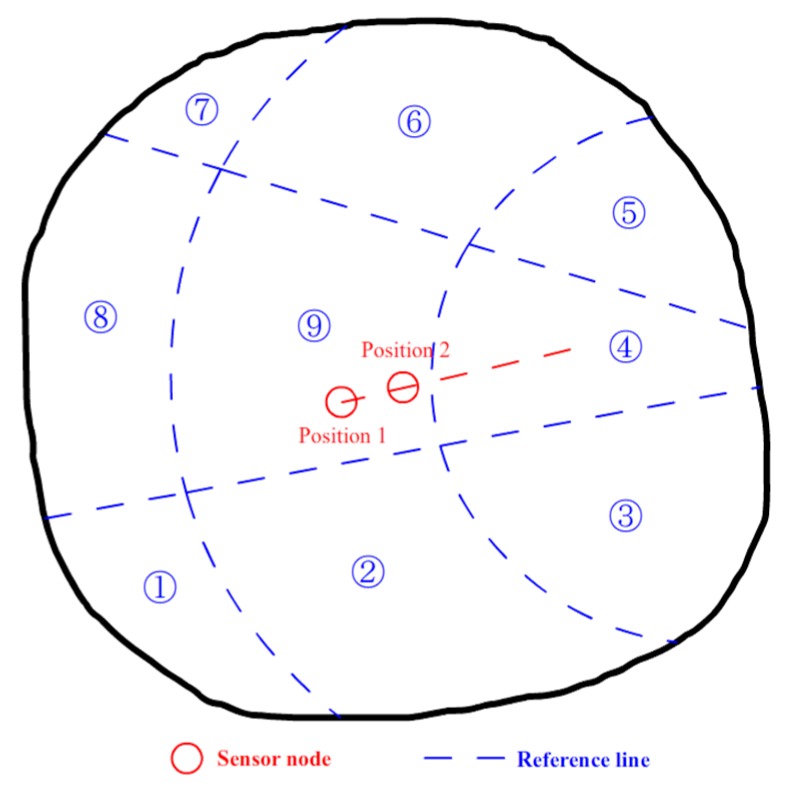
Illustration of the principle of node movement.

After the values of the entire BN are updated, the sensor nodes can move towards the area with higher intrusion probability. This procedure allows the system to focus attention on the area of concern and avoid wasting system resources on the safe areas. Figure 5 shows the principle of node movement. After adaptive learning at each round, the *P* values for the surrounding eight area pieces are compared to find the maximum value, *P*_max_. Then, if the *P_i_* value for the area that contains the sensor node is lower than *P*_max_, the sensor node moves towards the area with *P*_max_ according to the reference route from the node to the center of the destination. For example, in Figure 5, area ④ has the highest *P* value, so the sensor node in ⑨ should move towards the center of area ④ according to the red dotted reference line. The *P_i_* values are refreshed at each learning round when an intrusion happens. In order to avoid disorder, if an intrusion happens while there are sensor nodes which are still in moving status, the *P_i_* values are refreshed but not adopted immediately for execution. The new values are inserted into a queue according to their sequences. When the movement finishes and the current learning round is over, the new values are taken to work. Thus, there will be no disorder if *P*_max_ changes while nodes are still moving towards nodes with older *P*_max_. Moreover, the *P_i_* values are suggested to be accurate to two decimal places, and it is rare to meet two same *P*_max_ in the system. When there is more than one BN node with the same *P*_max_, the node with lowest serial number will be adopted. The moving distance is calculated by Equation (4), where *t* indicates the time count for moving towards this area piece, *ε* and *β* are parameters for adjusting the curve shape of the function, and *k* and *c* are parameters influencing the linear mapping of the equation. At each round of adaptive adjustment, a sensor node moves towards the destination until the distance *D* is reached. The intermittent movement will finally finish when the learning procedure is over. All those constant parameters, *ε*, *β*, *k* and *c*, in Equation (4) are used to influence the distance result of sensor node’s movement. They can be put together to consider. A larger moving distance at one time will lead the sensor node to the target faster but reduce the chance to revise the erroneous destination by subsequent learning round. Thus, the setting of these parameters is skillful and should be assigned by experienced engineer before the system runs according to the real application environment. Moreover, based on Equation (4), along with the increment of *t*, the movement distance will decrease gradually for a weakening accumulated effect of adjustment.

(4) $$D=\left[\frac{\varepsilon }{1+e^{-\beta t}}-\frac{\varepsilon }{1+e^{-\beta \left(t-1\right)}}\right]\cdot \left[k\cdot (P_{\max}-P_{i})+c\right]$$

**Figure 6:**
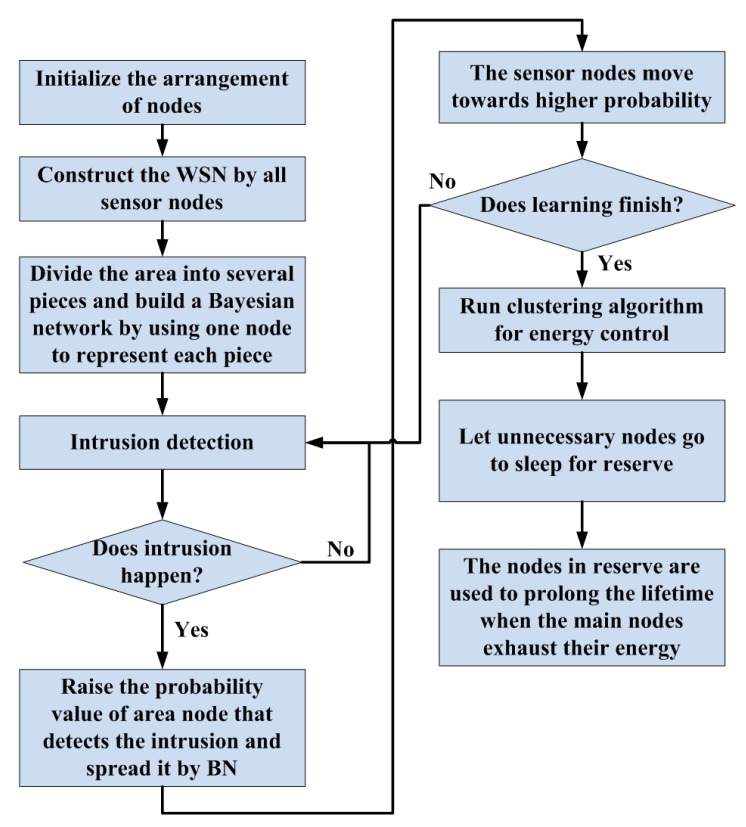
The procedure of adaptive learning and energy control.

The entire procedure of adaptive learning in this work is shown in Figure 6. In the first step, sensor nodes are initially uniformly arranged in the testing area, and the WSN is constructed among them. In the second step, the testing area is divided into several pieces, and the BN nodes are used to represent each of them. Then, the adaptive learning procedure is run to adjust the arrangement of sensing units. Once an intrusion occurs, the probability value of the intruded-on piece of the area be increased, and the surrounding region will also raise their guard level through a diffusional operation in the BN. After optimization of the BN, the sensor nodes will move towards the higher risk area to enhance the security. The system will repeat the learning procedure until it finishes. The learning end condition can be set following two ways in realization. In the first way, because an intrusion will trigger a learning round, a fixed intrusion time threshold is indicated by the engineer. If the intrusion time achieves this value, the learning procedure can be finished automatically. In the second way, the ratio of the total sensoring range to the area of the region is set as the threshold for learning procedure. The total sensoring range is estimated by the nodes’ locations and the detecting range of them eliminating the duplicate parts. For example, if the ratio threshold is set to 30%, the sensor nodes will move towards the key region gradually and duplicate their sensoring range until the total sensoring range only covers 30% of the whole detecting area. Thus, the threshold value in this way can adjust the balance between accuracy and breadth of detection. The specific values of the threshold in these two ways should be assigned by the engineer according to the application purpose and the environmental geography. The value will drastically influence the effect of the algorithm and so the assignment needs expertise. In the third step, the clustering algorithm and energy control method are adopted to prolong the lifetime of the system.

## 4. Data Fusion and Energy Control

Because there may be several sensor nodes in a common area in the testing region, it is necessary to employ a data fusion mechanism to negotiate their decision. The mechanism presented here follows the assumption that the sensor node nearer to the intrusive target has a higher probability of acquiring accurate results. Thus, they will have more weight in the voting for the final decision. Before making the decision, a clustering algorithm and target tracking are also needed to provide the relative information.

### 4.1. Clustering Algorithm

Due to the adaptive learning procedure mentioned above, the sensor nodes will gather in the key area with high intrusion probability. This action may directly lead to the redundancy of nodes in the area, and a decision-making mechanism should also be determined to acquire the final result according to all the reports from the nodes. Clustering is a good choice for this situation. All the sensor nodes are divided into several groups according to their positions on the map by the clustering algorithm. Then, the typical nodes are selected to represent others for energy savings, and the final decision can also be made through voting inside the cluster.

The *k*-means algorithm is a well-known partitional clustering algorithm that uses a squared-error-based optimization approach. In practice, as Equation (5) shows, the algorithm attempts to find a partition Φ such that the sum of the squared distances between patterns in each cluster and the respective representative element is minimized [24]:

(5) $$\underset{\Phi }{\arg\min}\sum_{i=1}^{k}\sum_{x_{j}\in C_{i}}{\left\|x_{j}-r_{i}\right\|}^{2}$$

where *r_i_* denotes the representative of the *i*-th cluster *C_i_*. Solving a *k*-means problem is computationally hard (NP-hard). However, Lloyd proposed efficient heuristics that quickly converge to a local optimum and are still widely used today [25]. Lloyd’s algorithm begins with *k* arbitrary centers, which are usually selected uniformly at random from input patterns. The algorithm then proceeds by alternating between the assignment and center calculation steps until the process stabilizes. In the assignment step, each point is assigned to the nearest center chosen before, and each center is recomputed as the center of mass of all points assigned to it in the center calculation step.

**Algorithm 1.** Center initialization of the *k*-means++ algorithm.**Input**: a set of objects *O***Output**: a set of initial centers $\hat{S}$, containing *k* elements**procedure** K-MEANS++ (*O*, *k*)   Set all the objects in *O* as unprocessed, and $\hat{S}\leftarrow \emptyset$;   Choose an initial center ${\hat{s}}_{1}$ randomly from the dataset, and $\hat{S}=\hat{S}\cup \left\{{\hat{s}}_{1}\right\}$
   **while**
$\left|\hat{S}\right|<k$       Choose the next initial center ${\hat{s}}_{i}$, selecting ${\hat{s}}_{i}=x\in O$ with probability $\frac{D{\left(x\right)}^{2}}{\sum_{x\in O}D{\left(x\right)}^{2}}$
       $\hat{S}=\hat{S}\cup \left\{{\hat{s}}_{i}\right\}$   **end while**   **return**
$\hat{S}$**end procedure**

In practice, the calculation process will always terminate, and few iterations are usually required, which makes it much faster than most of its competitors. However, its fast speed and simplicity come at the price of accuracy. It has been found that the *k*-means algorithm generates arbitrarily bad clustering in many natural examples. Thus, a *k*-means++ method is proposed to improve the technique by choosing a set of carefully selected initial centers instead of random initialization [26,27]. If we assume *D*(*x*) to be the distance of a sensor node *x* to its nearest center that is already selected, the initialization of *k*-means++ can be described by Algorithm 1. After its center initialization, *k*-means++ functions the same as the standard *k*-means algorithm to acquire the clusters. When the clusters are determined, the use of an energy control method is advised to reduce the redundant energy consumption. In this work, a representative coefficient *γ* = 1/*n*, *n* = 1, 2, … 10, is defined to arrange the representatives in a cluster. If *γ* = 1/3, one third of the sensor nodes in a cluster will be selected as representatives to work full time, while the others are put to sleep as reserves. This mechanism can reduce the energy consumption, and the reserved nodes are used to prolong the lifetime of the system when the representatives’ batteries are exhausted. Furthermore, in this work, it is suggested that the representatives be uniformly chosen from the queue according to the distance between the sensor node and cluster center. For example, in a cluster, nine sensor nodes are sorted as a queue numbered (1–9) according to their distance from the cluster center. Then, if *γ* = 1/3, the representatives can be chosen according to the number set {1,4,7}, {2,5,8} or {3,6,9} for uniformity. This mechanism will retain the balance in intrusion detection when conserving energy. If a representative exhausts its energy, the geographically nearest reserved sensor node should be activated to replace it and prolong the lifetime of the system.

### 4.2. Target Tracking

After the clustering of sensor nodes, the position of the intrusive target needs to be known for the data fusion to follow. Because there is only a localization device on a sensor node but no exact measurement equipment to acquire the distance from node to target, the location of the target can be only approximately estimated. To improve the accuracy of estimation, filter tools can be used to reduce the noise. The KF is a powerful tool that provides a recursive solution through a linear optimal filtering to estimate systems’ state variables [28]. Due to its low computational cost and good performance, it has been widely used in digital circumstances. A normal KF adopts a two-step process consisting of prediction and updating. In the prediction step, the current state variables ${\hat{x}}_{k}^{-}$ and the a priori estimate of their covariance $P_{k}^{-}$ are estimated by the filter. Subsequently, the a posteriori estimate state $\hat{x}$ along with the a posteriori estimate covariance $P_{k}$ are refreshed in the updating step based on the a priori estimates. However, the standard KF can be deployed only in a linear model. If the system is nonlinear, a linearization process needs to be applied to approximate the system with a linear time varying (LTV) system at each step. Using this approximation leads to a new filtering tool, the EKF [29,30]. As shown in Equations (6) and (7), these two stochastic differential equations are used to describe the nonlinear process.

(6) $$x_{k}=f(x_{k-1},u_{k-1},w_{k-1})$$

(7) $$z_{k}=h(x_{k},v_{k})$$

where *x_k_* is the process state at time *k*; *w_k_* represent the excitation noise; *v_k_* denotes the observation noise; and *z_k_* and *u_k_* represent the observed variable and the control vector at time *k*, respectively.

An EKF estimation procedure generally includes four steps: initialization, linearization, prediction and updating. In the initialization step, the values for ${\hat{x}}_{k}^{-}$ and $P_{k}^{-}$ are initialized for further estimation, where the symbol ‘^’ denotes the estimation value and the symbol ‘−’ represents the a priori element. Following initialization, an approximate linear process can be built by the linearization step for the nonlinear system. Equations (8) and (9) are then introduced to estimate the a priori value of *x* in the predication step.

(8) $${\hat{x}}_{k}^{-}=f({\hat{x}}_{k-1},u_{k-1},0)$$

(9) $$P_{k}^{-}=A_{k}P_{k-1}A_{k}^{T}+W_{k}Q_{k-1}W_{k}^{T}$$

where the symbol ${\hat{x}}_{k}^{-}$ and $P_{k}^{-}$ denote the estimate of *x* and the a priori estimate covariance at time *k −* 1, given observations up to and including time *k* − 1, respectively; the notation $A_{k}$ represents the state transition model and *Q_k_* denotes the covariance of the process noise; and *W_k_* and *u_k_* represent the Jacobian matrix of the process and the control vector at time *k*, respectively.

Then, the a posteriori state estimate of *x* can be refreshed at the update step based on the a priori estimate according to Equations (10)–(12). The new posteriori state estimate lies between the predicted and measured states, and it has better estimated uncertainty than either of them. For each round, the prediction and update steps are repeated to create the new estimate and its covariance, and these values are applied to the next iteration. Thus, only the last estimate value is required by the EKF to calculate a new state. The relevant equations are as follows:

(10) $$P_{k}=(I-K_{k}H_{k})P_{k}^{-}$$

(11) $$K_{k}=P_{k}^{-}H_{k}^{T}{\left(H_{k}P_{k}^{-}H_{k}^{T}+V_{k}R_{k}V_{k}^{T}\right)}^{-1}$$

(12) $${\hat{x}}_{k}={\hat{x}}_{k}^{-}+K_{k}(z_{k}-h({\hat{x}}_{k}^{-},0))$$

where ${\hat{x}}_{k}$ represents the a posteriori state estimate; $P_{k}$ denotes the a posteriori estimate covariance at time *k* given observations up to and including time *k*; *K_k_* represents the Kalman gain at time *k*; *H_k_* and *V_k_* denote the Jacobian matrices of the observations for mapping the true states onto the observed ones; and *z_k_* and *R_k_* represent the observation of the true state *x_k_* and the covariance of the observation noise, respectively.

### 4.3. Data Fusion

Due to the adaptive learning procedure, the mobile nodes gather in the key area with high intrusion probability, which constitutes a decision-making problem: When several sensor nodes in a common area acquire different detection results simultaneously, which one will be followed? A data fusion method needs to be designed to negotiate the reports from each node and make a final decision. According to the assumption that sensor nodes nearer to the target have a higher probability of obtaining accurate reports, higher weights are assigned to these nodes in the negotiation procedure. After the clustering and adaptive moving, the positions of mobile sensor nodes are certain before each decision. Because there is no distance-measuring equipment, only GPS devices, on the mobile sensor nodes, it is hard to know the exact position of the intrusive target, but the distance between two sensor nodes can be calculated. Thus, the coordinate of the sensor node nearest to the target in the map is considered to indicate the location of the target.

The nearest node is judged according to the last estimated position of the target and the EKF algorithm. Then, the decision is made based on the cluster of sensor nodes nearest to the intrusive target, and the voting decision value *E* can be computed following Equation (16), where *k* indicates the number of nodes in the cluster, *ω_i_* denotes the voting weight of node *i*, and *B_i_* is the result report from node *i*, which is a Boolean value of either 0 or 1. First, the distance between node *i* and the node nearest to the intrusive target is obtained by Equation (13). Then, the distance *d_i_* is normalized by Equation (14), where *d*_max_ represents the maximum distance from the node nearest to the target in a common cluster. Finally, the voting weight *ω_i_* can be calculated through Equation (15), where a standard Gaussian distribution is used to map the distance value to a voting weight and *μ* and *σ* are parameters of the distribution, as shown in Figure 4b; *δ* is an adjustment coefficient assigned by the user according to the application. We suggest that engineers should choose a suitable *δ* value to make sure that the value of voting weight *ω_i_* is less than or equal to 1 after adjusting. For example, if the parameter *μ* = 0, *σ* = 0.5, the maximum value of function is 0.8 as shown in Figure 4b. Thus, *δ* should be set *δ* ≤ 1.25 to make *ω_i_* ≤ 1. Furthermore, the *δ* is commonly related to the *E_thr_* value, a small *δ* is suggested to work with a low *E_thr_* value. After obtaining the decision value *E*, the final decision can be made by a threshold *E_thr_*. If *E* > *E_thr_*, the cluster is considered to be a finder of an intrusive target; otherwise, there is no target to be found. *E_thr_* is a decision threshold for data fusion. Its task is to control the balance between detection rate and accuracy. A higher *E_thr_* value requires more evidence to prove the entrance of target and will increase the accuracy. However, it may miss some real intrusions with noise and that will reduce the detection rate. On the contrary, a lower *E_thr_* allows higher detection rate with lower accuracy. In conclusion, *E_thr_* value should also be defined by the user according to the application environment. If the system needs high accuracy, the larger *E_thr_* should be adopted; otherwise, the smaller one may be suitable. The relevant equations are as follows:

(13) $$d_{i}=\sqrt{{\left(x_{i}-x_{nearest}\right)}^{2}+{\left(y_{i}-y_{nearest}\right)}^{2}}$$

(14) $$d_{i-norm}=d_{i}/d_{\max}$$

(15) $$\omega_{i}=\delta \cdot (1/\sqrt{2\pi }\sigma )e^{-\frac{{\left(d_{i-norm}-\mu \right)}^{2}}{2\sigma^{2}}}$$

(16) $$E=\sum_{i=1}^{k}\omega_{i}\cdot B_{i}/k$$

**Figure 7:**
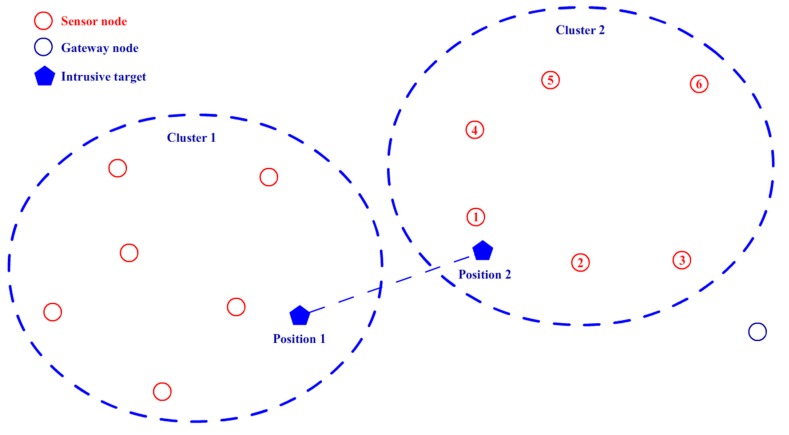
Example of the data fusion procedure.

Figure 7 shows an example of the data fusion procedure. After the clustering, Cluster 1 and Cluster 2 each contain six sensor nodes. The intrusive target moves from position 1 to position 2. Because there are no distance-measuring devices on any of the sensor nodes, the system cannot obtain the exact position of the target; it can obtain only the node position nearest to the target. According to the relative position between the two clusters and the target movement direction estimated by EKF as mentioned above, node 1 in Cluster 2 is estimated to be the nearest sensor node that may first detect the target when it has just entered the detection range of Cluster 2. Then, the coordinate of the target is denoted by the position of node 1, and the data fusion procedure is run following Equations (13)–(16). Finally, a decision is made via the voting decision value *E* and its threshold *E_thr_*.

## 5. Simulation and Experiments

### 5.1. Simulation Results and Discussion

To verify the effects of the adaptive target detection (ATD) system, simulation is performed in MATLAB on a PC with a 3.4-GHz Intel Core CPU and 4 GB of memory. In the simulation, sensor nodes are uniformly deployed in a 400 m *×* 400 m rectangular field in the initial step. The field has four roads from southern, northern, eastern and western directions to the center. There are four different algorithms involved in the simulation: the 2D 2-barrier (2DB) method [10], collaborative target detection (CTD) [4], static target detection (STD) and ATD, which has been introduced in the current study. The 2DB algorithm has the ability to catch intruders who enter from one side and try to cross the field, and its rotation mechanism for sensor nodes can help the system conserve energy [10]. CTD uses data fusion to acquire a reliable final decision [4]. In CTD, the sensor nodes report their raw measurements or binary decisions at a local area to the fusion center, and the center collects the data reported in consecutive periods to guarantee the quality of the final detection results. STD uses the initial arrangement of ATD without an adaptive learning procedure, data fusion and energy control. It reports the intrusion immediately once it detects the intrusive target without negotiation between nodes. However, ATD should employ the data fusion procedure to improve the accuracy.

The simulations are run in two different modes. The directional intrusion mode allows the targets to enter the area only from the four directional roads, while the random mode allows the targets to intrude from a random direction, which is often chosen by a random number generated by a computer program. For the detection accuracy and energy consumption test, the speed of intruders is set to 10 m/s, the sensing range of nodes is 20 m, and the communication range is set to 100 m. In the initial step of ATD, the interval parameter *R_int_* is set according to the number of nodes participating in the simulation. When the number is 200, *R_int_* is set to 3, *D*_2_ = 2*D*_1_, and *D*_3_ = 2*D*_2_. If the number is 400, *R_int_* is set to 4, *D*_2_ = 2*D*_1_, *D*_3_ = 2*D*_2_, and *D*_4_ = 2*D*_3_. If there are 800 sensor nodes involved in the test, *R_int_* is set to 5, *D*_2_ = 2*D*_1_, *D*_3_ = 2*D*_2_, *D*_4_ = 2*D*_3_, and *D*_5_ = 2*D*_4_. The BN structure is built by concentric circles and two reference straight lines at 60° and 120° following the recommended method as shown in Figure 2b. The decision threshold *E_thr_* is set to 0.6, and *δ* = 1.5 and *μ* = 0 and *σ* = 0.5 in Equation (15). Furthermore, the clustering parameter of ATD, *γ*, is set to 1/3, *η* = 2, *ΔP_i_* = 0.5 in Equations (1) and (2), *α* = 0.5 in Equation (3), and *ε* = 5, *β* = 0.5, *k* = 10, and *c* = 3 in Equation (4). For the BN structure, the parameter *λ* in Figure 3 is set to 1/2*^n^*, where *n* indicates the distance between the corresponding area node and the area node containing the target. The ATD system is trained 150 times to adjust the position of the mobile sensor nodes before the detection test. After the training procedure, the nodes cannot be moved further, and no additional energy costs will be incurred by movement.

**Figure 8:**
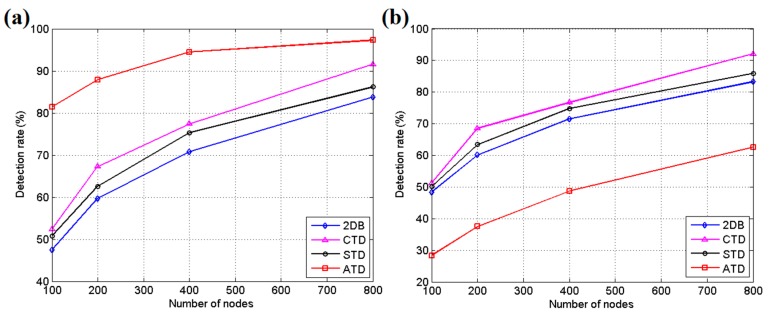
(**a**) Average detection rate for directional intrusion; and (**b**) average detection rate for random intrusion.

**Figure 9:**
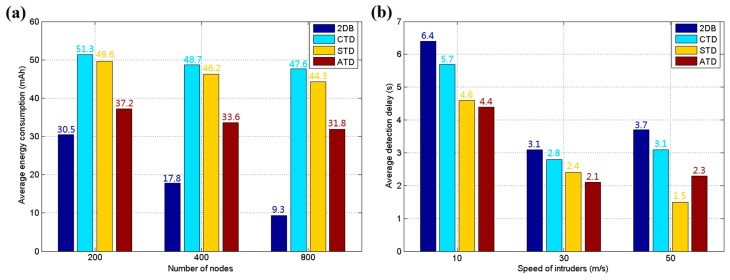
(**a**) Average energy consumption of each sensor node in an hour; and (**b**) average detection delay for each method.

Figure 8 shows the simulation results for 100, 200, 400 and 800 sensor nodes in the area in these two different modes. Three hundred simulation tests are run for each group, and the average results are calculated for presentation. As shown in Figure 8a, in the directional mode, ATD achieves the highest detection rate, which means the correct detection without misjudgment in the test. Its advantage is more easily discerned when the number of sensor nodes is lower due to its aggregation effect of nodes for the key position. However, this scenario will directly lead to poor performance in random mode, as shown in Figure 8b, because there are not enough sensor nodes to cover all possible areas. The 2DB approach exhibits relatively poor performance in these two modes because it allows only one band of sensors within each grid wake up, with the others going to sleep to conserve energy. 2DB can usually detect the objects that cross the field, but it is insensitive to irregular motion. However, 2DB involves less energy consumption than the other methods, as indicated in Figure 9a.

The overall energy consumption simulation results are shown in Figure 9a for 200, 400 and 800 sensor nodes. The total energy used by the system is divided by the number of nodes and running time to acquire the average energy consumption for each node per hour. It was found that the CTD and STD methods used more energy than ATD due to the lack of an energy control mechanism, and the data fusion step of CTD costs additional energy. Figure 9b shows the results of the system response speed test, in which the intruders enter the area with different speeds. The average detection delay reflects the real-time ability of the WSN detection system. It can be found that the ATD algorithm introduced in this study has satisfactory real-time response, which is important for the system to deal with intrusion. CTD requires slightly more time to complete the negotiation, and 2DB requires a moment to wake up its sleeping nodes. STD has the lowest reaction time when a target enters at a speed of 50 m/s due to its direct report without confirmation, which leads to low accuracy in detection. In summary, according to the simulation results, ATD holds the advantage in intrusion detection for directional targets, with good performance for reaction speed and energy control. However, it is not suitable for an area that can be intruded upon from many random directions. Because there are many scenarios with only directional paths, such as gardens or playgrounds, ATD can be employed to avoid wasting system resources on large secure areas with low probability of being intruded upon.

**Figure 10:**
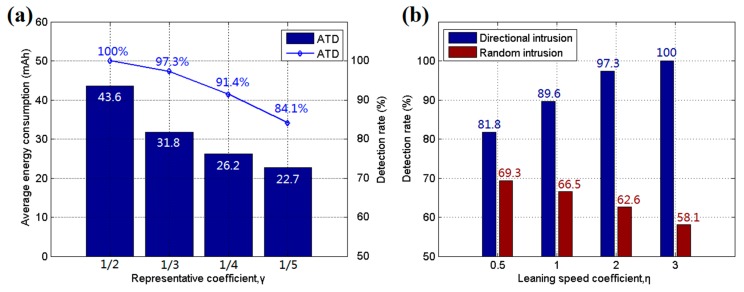
(**a**) Average energy consumption and detection rate for different representative coefficients *γ*; and (**b**) detection rate for different learning speed coefficients *η*.

Several parameters need to be set by the user for ATD, and these can influence the performance of the algorithms. The simulation is run to evaluate the effect of these parameters, and other environmental variables are set the same as in the accuracy tests. Figure 10a shows the influence of different representative coefficients *γ* in the clustering algorithm. A decrement in *γ* causes more sensor nodes to go to sleep to conserve energy. It was found that a low *γ* value reduces the detection rate of the system but conserves more energy, prolonging the system’s life. Thus, a suitable *γ* value should be chosen by the user according to the main purpose of the system. Figure 10b shows the influence of another important coefficient, *η*, in Equations (1) and (2). The test was run with 800 sensor nodes and the same environmental variables as in the accuracy tests, and the detection rates for directional and random intrusions were recorded. The learning speed coefficient *η* affects the probability influence of an intrusive target towards the area node in the BN network. A high *η* increases the gathering effort of the MWSN and increases the ability of the system to react to directional intrusion. However, this enhancement weakens its detection ability for random intrusions. Thus, the values should also be assigned by the user according to the application environment.

### 5.2. Implementation and Experiments

**Figure 11:**
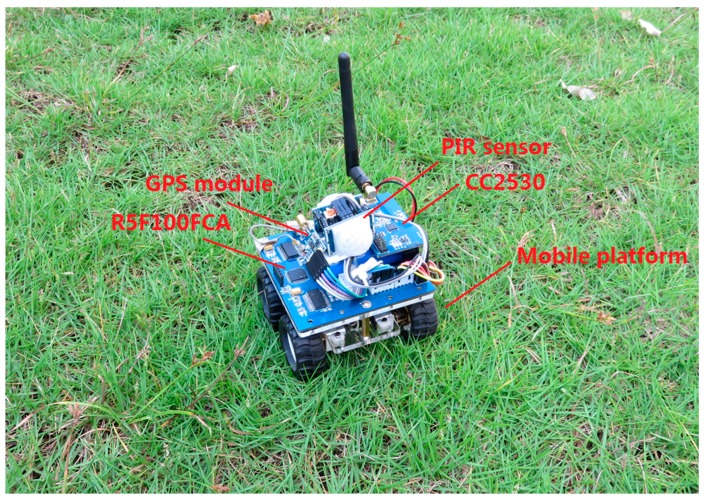
Illustration of the mobile sensor node for experiments.

**Figure 12:**
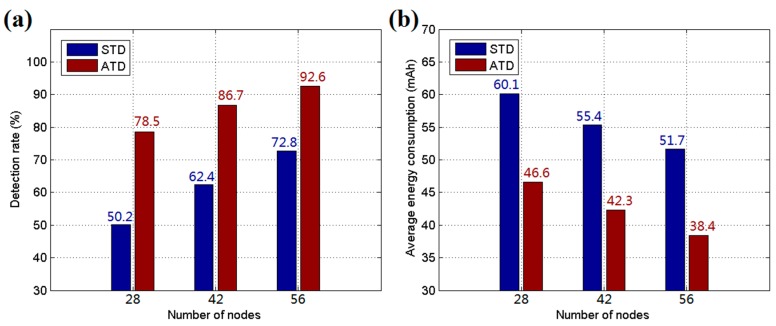
(**a**) Average detection rate for experiments; and (**b**) average energy consumption of each sensor node per hour for experiments.

As shown in Figure 11, to further verify the effect of ATD, the hardware of a WMSN node was implemented on the Renesas R5F100FCA and TI CC2530 platforms. The R5F100FCA is a 16-bit microcontroller and is used to drive the detection device and mobile platform. The CC2530 is a Zigbee (IEEE 802.15.4) system-on-chip (SOC) used to establish the WSN in the detection system. Two PIR sensors are employed in the hardware to detect intrusive targets from all directions. Moreover, a GPS module is adopted to acquire the position of the node, and the device can move via a mobile platform with four wheels driven by DC motors. The experiments were performed in a 200 m × 200 m rectangular field of a park. In reality, the detection range of the node is more than 20 m, and the communication range exceeds 100 m. ATD was employed in the experiments with *γ* = 1/3, *η* = 2, and Δ*P_i_* = 0.5 in Equations (1) and (2); *α* = 0.5 in Equation (3); and *ε* = 5, *β* = 0.5, *k* = 10, and *c* = 3 in Equation (4). The decision threshold *E_thr_* was set to 0.6, and *δ* = 1.5, *μ* = 0, *σ* = 0.5 in Equation (15). The BN structure was initialized following the recommended method. The parameter *R_int_* was set to 3, *D*_2_ = 2*D*_1_, and *D*_3_ = 2*D*_2_. The speed of intruders was set to 5 m/s, and the numbers of sensor nodes for three testing groups were set to 28, 42, and 56, respectively. For each group, ATD was trained 50 times to adjust the positions of the sensor nodes before formal tests. After the training procedure, the nodes were fixed, and the targets were allowed to enter for detection. The experiments were run twenty times, and the average detection rate results are shown in Figure 12a. The average energy costs are recorded and calculated for each node per hour, as shown in Figure 12b. From the experimental results, it was found that ATD offers better performance than STD due to its adaptive learning procedure and energy control mechanism. Moreover, ATD is suitable for use under circumstances in which the targets intrude along several certain pathways.

## 6. Conclusions

Security is a basic need in daily life. A detection system for abnormal objects or events in regions of interest based on electronic technology is able to satisfy this demand. However, because the chance of being intruded upon is not equal for each region of a certain area, traditional fixed observing stations or a static sensor network cannot accommodate various application environments well. Static stations or nodes without any moving equipment cannot adjust their position and are instead arranged manually before system operation. Thus, after setup, they are likely to miss targets due to unsuitable initial positions. Mobile WSNs are suitable for this situation and have the ability to improve the adaptation and capability of the system. The adaptive leaning procedure adopted in this work can lead mobile sensor nodes to move towards the key area, and the EKF is used to increase the localization accuracy of targets. Real-time hardware was implemented on the Renesas R5F100LEA MCU platform with a TI CC2530 Zigbee chip for communication. The simulation and experimental results demonstrate the improvements achieved by the new system.
